# The Essentiality of Staphylococcal Gcp Is Independent of Its Repression of Branched-Chain Amino Acids Biosynthesis

**DOI:** 10.1371/journal.pone.0046836

**Published:** 2012-10-04

**Authors:** Ting Lei, Junshu Yang, Li Zheng, Todd Markowski, Bruce A. Witthuhn, Yinduo Ji

**Affiliations:** 1 Department of Veterinary and Biomedical Sciences, College of Veterinary Medicine, University of Minnesota, St. Paul, Minnesota, United States of America; 2 College of Biological Sciences, University of Minnesota, St. Paul, Minnesota, United States of America; University of Edinburgh, United Kingdom

## Abstract

Our previous studies revealed that the staphylococcal protein Gcp is essential for bacterial growth; however, the essential function of Gcp remains undefined. In this study, we demonstrated that Gcp plays an important role in the modulation of the branched-chain amino acids biosynthesis pathway. Specifically, we identified that the depletion of Gcp dramatically elevated the production of key enzymes that are encoded in the *ilv-leu* operon and responsible for the biosynthesis of the branched-chain amino acids isoleucine, leucine, and valine (ILV) using proteomic approaches. Using qPCR and promoter-*lux* reporter fusions, we established that Gcp negatively modulates the transcription of the *ilv*-*leu* operon. Gel-shift assays revealed that Gcp lacks the capacity to bind the promoter region of *ilv.* Moreover, we found that the depletion of Gcp did not influence the transcription level of CodY, a known repressor of the *ilv-leu* operon, while induced the transcription of CcpA, a known positive regulator of the *ilv-leu* operon. In addition, the depletion of Gcp decreased the biosynthesis of N^6^-threonylcarbamoyladenosine (t6A). To elucidate whether the essentiality of Gcp is attributable to its negative modulation of ILV biosynthesis, we determined the impact of the *ilv*-*leu* operon on the requirement of Gcp for growth, and revealed that the deletion of the *ilv*-*leu* operon did not affect the essentiality of Gcp. Taken together, our results indicate that the essentiality of Gcp isn’t attributable to its negative regulation of ILV biosynthesis in *S. aureus*. These findings provide new insights into the biological function of the staphylococcal Gcp.

## Introduction

*Staphylococcus aureus* is an important pathogen that can cause severe human and animal infections. The prevalence of multi-drug resistant *S. aureus*, especially methicillin- and vancomycin-resistant *S. aureus* has caused serious public health concerns [1], [2]. Understanding the physiology of bacterial cells allows us to identify alternative strategies to combat *S. aureus*. Our previous studies have demonstrated that a Gcp is required for the viability of *S. aureus* during *in vitro* culture [3] and is involved in the modulation of bacterial autolysis [4]. However, the essential function of Gcp remains undefined.

The metabolic pathway of the branched-chain amino acids (BCAAs) isoleucine, leucine, and valine (ILV) plays an important role in bacterial physiology. These hydrophobic amino acids are crucial for maintaining protein structure and function [5]. The branched-chain alpha-keto acids, both the first catabolic and the last anabolic intermediates of ILV, are the precursors of the branched-chain fatty acids that are the major fatty acids involved in bacterial cell membrane biosynthesis [6], [7]. Moreover, the immediate precursor of valine, α-ketoisovalerate, is the precursor of leucine and involved in the synthesis of cofactors pantothenate and coenzyme A [8]. The biosynthesis of BCAAs requires several key enzymes. In *Bacillus subtilis*, the genes encoding these enzymes are well characterized, including the *ilvBHC*-*leuABCD* (*ilv-leu*) operon and *ilvA*, *ilvD*, *ybgE* and *ywaA* genes distributed around the genome [9], [10], [11]. In *S. aureus*, there is a similar *ilv*-*leu* gene cluster *ilvDBHC*-*leuABCD*-*ilvA* containing 9 genes that are involved in the ILV biosynthesis pathway. Another gene *ilvE* encoding an aminotransferase has a similar function with *ybgE* and *ywaA* of *B. subtilis*.

Several mechanisms of regulating the biosynthesis of BCAAs have been revealed and characterized in different organisms. In *B. subtilis*, it has been shown that a T-box anti-termination system mediates the *ilv*-*leu* operon in response to leucine availability [9], [12]; moreover, the specific cleavage sites of the full-length transcript and the stability of the cleavage product affect the transcription level of each gene in the *ilv*-*leu* operon [13]. In *S. aureus*, it was revealed that a global regulator CodY represses the BCAA biosynthesis [14]; whereas two global regulators of nitrogen metabolism in *B. subtilis*, CodY and TnrA, repress the BCAA biosynthesis through binding to an upstream regulatory region of the *ilv-leu* operon [14], [15], [16], [17], [18]. In addition, in *B. subtilis* a major regulator of carbon metabolism, CcpA, positively regulates the transcription of the *ilv*-*leu* operon by binding to the promoter region [10], [18], [19]. Although *S. aureus* possesses all genes necessary for biosynthesis of BCAAs, the bacterium exhibits an auxotrophic phenotype for BCAAs through an unknown mechanism [20], [21].

Recent biochemical analysis from *E. coli* showed that Gcp homologues are involved in the biosynthesis of N^6^-threonylcarbamoyladenosine (t6A) and tRNA modification [22], which is a highly conserved mechanism. In this study, we identified that the BCAA biosynthesis pathway and the biosynthesis of N^6^-threonylcarbamoyladenosine (t6A) are controlled by the essential protein Gcp and demonstrated that Gcp modulates the transcription of the *ilv*-*leu* operon in *S. aureus*. The elimination of isoleucine, leucine, and valine remarkably enhanced bacterial growth during the depletion of Gcp. Importantly, we found the essentiality of Gcp is not attributable to the negative modulation of BCAA biosynthesis. These new findings provide new insights into the biological function of the essential protein, Gcp.

## Results

### Identification of IlvA, IlvB and IlvD Proteins that are Elevated by the Depletion of Gcp Using Proteomic Approaches

**Figure 1:**
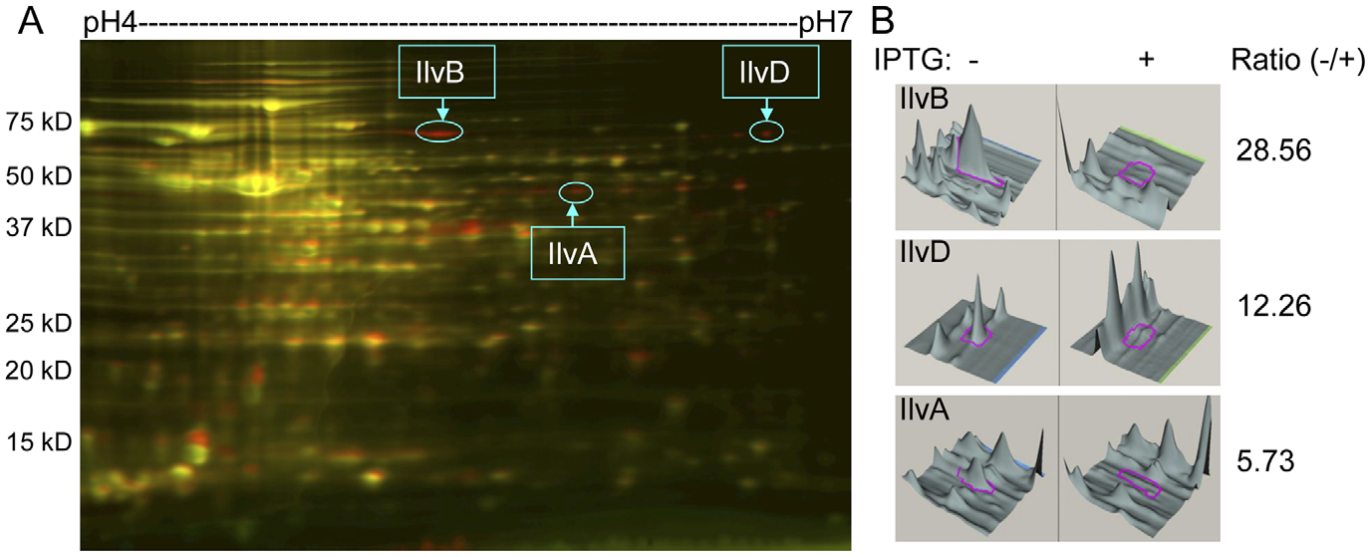
Effect of the down-regulation of Gcp on global gene expression. (A) 2D-DIGE analysis of the impact of the down-regulation of Gcp on global gene expression in the culture of the *Pspac*-regulated *gcp* expression strain (JRN0105), the arrows indicate the identity of proteins in the selected protein spots. (B) 3D review of the selected protein spots, and the changes of the selected protein spots containing IlvA, IlvB, and IlvD, respectively, during the depletion of Gcp.

In order to explore the potential function of the essential protein Gcp, we examined the impact of Gcp on global protein production of *S. aureus* by using 2D-Differential In Gel Electrophoresis (DIGE) coupled with mass spectrometry. The bacterial cells were harvested from the mid-log phase cultures of the *Pspac*-regulated *gcp* expression strain with and without addition of inducer, IPTG as described [3]. The bacterial cells were lysed, and whole cell lysates was labeled with Cy2, Cy3 and Cy5 respectively as described in the Materials and Methods. The same amount of labeled proteins was loaded onto gradient SDS-PAGE following electrophoresis. Proteins with specific dyes from different samples were separated and compared. Ten differential spots were picked and followed by in-gel digestion and MALDI mass spectrometry. Based on the data obtained from the Scaffold database, three proteins were found more than once, including threonine dehydratase (IlvA, EC: 4. 3. 1. 19), acetolactate synthase (IlvB, EC: 2. 2. 1. 6), and dihydroxy-acid dehydratase (IlvD, EC: 4. 2. 1. 9) (Fig. 1A). The genes encoding the three proteins are located in an *ilv*-*leu* operon and are key enzymes for the biosynthesis of the branched-chain amino acids (BCAAs), isoleucine, leucine, and valine. Moreover, each pair of protein spots (Cy3 and Cy5-labeled) was further assessed in 3D views and statistically analyzed. We found that the expression of IlvA, IlvB and IlvD was significantly increased during the depletion of Gcp (Fig. 1B). The identity of remaining protein spots was not determined. The above results indicate that the essential staphylococcal Gcp may be involved in the modulation of the biosynthesis pathway of BCAAs.

### The Depletion of Gcp Dramatically Increased the Transcription of the *ilv-leu Operon*

**Figure 2:**
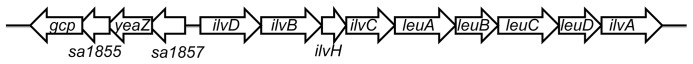
Illumination of the genomic context and arranges of *gcp* and *ilv*-*leu* operons.

Bioinformatics analysis of the *S. aureus* genome revealed that nine genes are sequentially aligned in the *ilvDBHC*-*leuABCD*-*ilvA* operon, which is similar to the *ilv*-*leu* operon responsible for the biosynthesis of the branched-chain amino acids in *B. subtilis* [11]. Genome mapping showed that the *gcp* operon (20) and the *ilv*-*leu* operon were juxtaposed on the genome of *S. aureus* (Fig. 2). To confirm the effect of Gcp on the expression of IlvA, IlvB, and IlvD, we examined the impact of the depletion of Gcp on the transcription of *ilvD* and *leuA* located on the *ilv*-*leu* operon, and *ilvE* gene, which is located elsewhere in *S. aureus* genome and encodes an aminotransferase. The qPCR results showed that the down-regulation of Gcp had no impact on *ilvE* RNA levels; whereas the RNA levels of *ilvD, leuA* and *ilvA* were elevated by approximately 5- to 111- fold, suggesting that Gcp mediates the transcription of the *ilv*-*leu* operon (Table 1). Interestingly, we found that the depletion of Gcp increased the transcription of *ccpA* (Table 1), which encodes CcpA, a possibly positive regulator of the *ilv*-*leu* operon.

**Table 1:** Gene transcription change due to the depletion of Gcp.

ORF (N315)	Gene	Fold Change^a^
*Sa0512*	*ilvE*	0.97
*Sa1098*	*codY*	0.78
*Sa1557*	*ccpA*	3.24
*Sa1858*	*ilvD*	5.45
*Sa1862*	*leuA*	68.59
*Sa1866*	*ilvA*	111.37

a The fold change represents the transcription levels of genes with the depletion of Gcp compared with those during the induction of *gcp* transcription with IPTG (200 μM) at exponential phase of growth (OD600 nm ∼0.5).

**Figure 3:**
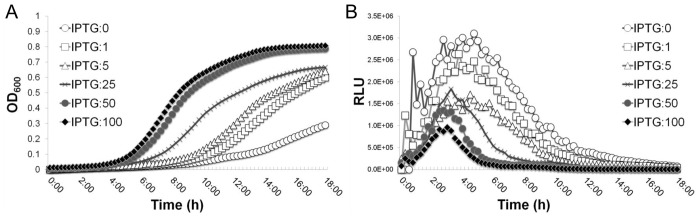
Effect of the down-regulation of Gcp on the *ilv* promoter transcription activity. (A) The growth curves of the *Pspac*-regulated *gcp* expression mutant containing the *ilv* promoter-*lux* reporter fusion (JRN0110) at the presence of different concentrations of inducer IPTG. (B) Relative Bioluminescence intensity of JRN0110 during the culture with different concentrations of IPTG (0, 1, 5, 25, 50 and 100 µM). The optical density at OD600 nm and bioluminescence intensity of cultures were simultaneously measured every 15 min for 18 hours at 37°C using a BioTek Synergy plate reader. The experiments were repeated at least five times. These figures are one representative of three independent experiments.

To further confirm the transcriptional regulation, we constructed an *ilv* promoter-*lux* reporter fusion in the *Pspac-*regulated *gcp* expression mutant. This system allows us to effectively down-regulate *gcp* expression and simultaneously to monitor the reporter gene expression by measuring the bioluminescence intensity [23]. The results showed that bacterial growth is dependent on IPTG, indicating the essentiality of Gcp (Fig. 3A); whereas the bioluminescence intensity was increased from the early log to mid-log phases of growth during the depletion of Gcp in a dose dependent manner (Fig. 3B). The *Pspac-*regulated *gcp* expression mutant carrying a promoterless *lux* reporter was utilized as a control. The addition of IPTG had no influence on the *lux* expression in the control strain (data not shown). Taken together, these data indicate that Gcp transcriptionally mediates the expression of the *ilv-leu* operon.

### The Recombinant Gcp Lacks the Capacity of Binding to the Promoter Region of *ilv*-*leu* Operon

Structural analysis of Gcp protein revealed that Gcp lacks a helix-turn-helix DNA-binding domain, suggesting that Gcp may indirectly regulate the transcription of *ilv*-*leu* operon. To further determine the mechanism of Gcp’s involvement in modulating the expression of the *ilv*-*leu* operon, we expressed and purified His-tagged Gcp protein as described [3] and performed gel-shift assays to examine whether Gcp binds to the promoter region of *ilv*. Negative controls included the labeled *ilv* promoter region without protein and the labeled *ilv* with BSA protein. The non-labeled *ilv* promoter region was used as a specific competitor, while an internal *ilv* fragment probe was used as a nonspecific binding control. The results showed that the addition of Gcp in the reaction mixtures containing the *ilv* promoter probe had no impact on the electrophoretic mobility of *ilv* probe compared to the controls (data not shown). This suggests that the regulation of the *ilv*-*leu* operon transcription by Gcp may not function through its binding to the *ilv* promoter region.

### Gcp is Involved in the Biosynthesis of N^6^-threonyl Carbamoyladenosine (t6A)

**Figure 4:**
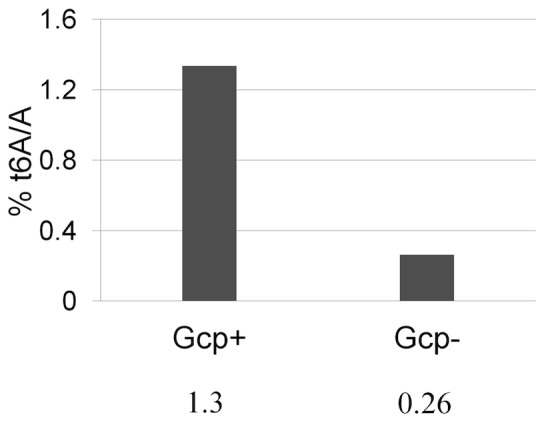
Determine the effect of depletion of Gcp on t6A content of tRNA isolated from staphylococcal cells using LC-MS/MS. tRNA isolated and purified from the defined *Pspac*-regulated *gcp* expression mutant with (Gcp+) and without inducer IPTG (200 μM) (Gcp-) at exponential phase of growth (OD600nm ∼0.5) and processed for LC-MS/MS analysis of t6A as described in Materials and Methods. The t6A content was normalized to the respective adenosine content and represented to % t6A/A ratio (the numerical values).

It has been reported that the Gcp homolog YgjD in *E. coli* is required for the synthesis of t6A [22]. To determine whether Gcp is involved in t6A biosynthesis in *S. aureus*, we quantitatively measured the t6A/A ratio in tRNAs purified from the staphylococcal cells under Gcp deplete (-IPTG) or Gcp-replete (+ IPTG) conditions using LC-MS/MS. When the defined *Pspac*-regulated *gcp* expression mutant was grown in TSB with IPTG, the ratio of t6A/A was 1.3%, whereas during depletion of Gcp by growing the mutant in the absence of IPTG the ratio of t6A/A observably decreased 5-fold to 0.26% (Fig. 4). This indicates that the staphylococcal Gcp is important for t6A modification.

### Down-regulation of *gcp* Enhances Bacterial Growth in ILV Dropout Chemically Defined Media

**Figure 5:**
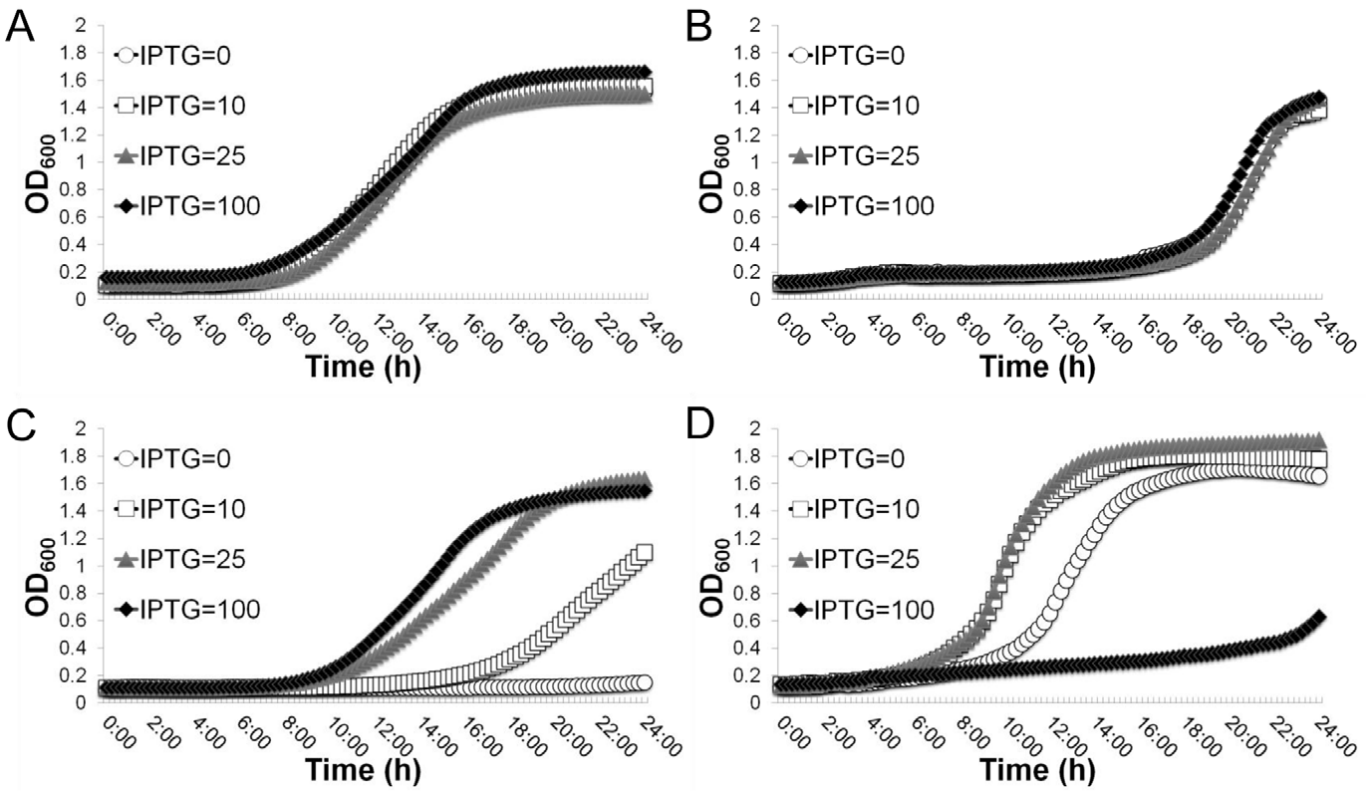
Effect of the depletion of the branched-chain amino acids isoleucine, leucine and valine (ILV) on the growth of the defined Pspac-regulated gcp expression strain. The growth curves of the wild type control, WCUH29 carrying pYH4-lacI in in nutrient complete CDM (A) and ILV dropout CDM (B); the growth curves of the defined Pspac-regulated gcp expression strain (JW290111) in in nutrient complete CDM (C) and ILV dropout CDM (D). The bacterial growth in the presence of different concentrations of inducer IPTG (0, 10, 25 and 100 µM) was monitored by kinetically measuring the optical density at OD600 nm every 15 min for 24 hours at 37°C using a BioTek Synergy plate reader. These figures are one representative of three independent experiments.

The above findings led us to hypothesize that the tight control of the branched-chain amino acids biosynthesis by Gcp may contribute to the essentiality of Gcp for growth. To test this possibility, we determined the effect of isoleucine, leucine and valine (ILV) on the requirement for Gcp by monitoring the growth of the defined *Pspac*-regulated *gcp* expression mutant in ILV dropout CDM with varying amounts of IPTG. The conditional *gcp* expression mutant and the wild-type control were inoculated into nutrient complete CDM and ILV dropout CDM, respectively. The wild-type control strain exhibited consistent growth curves regardless of IPTG concentrations in all growth media (Fig. 5A and 5B). A longer lag phase was seen in ILV dropout CDM (Fig. 5B) compared with nutrient complete CDM (Fig. 5A). However, in nutrient complete CDM the defined *Psapc*-regulated *gcp* expression mutant exhibited IPTG dependent growth; without IPTG induction bacterial growth was dramatically inhibited, indicating the essentiality of Gcp for growth (Fig. 5C). In contrast, in ILV dropout CDM, without IPTG induction the bacteria exhibited a robust growth phenotype; whereas in the presence of 100 µM IPTG bacterial growth was noticeably inhibited (Fig. 5D). In addition, IPTG dependent growth only occurred between 0 to 25 µM IPTG (Fig. 5D).

**Figure 6:**
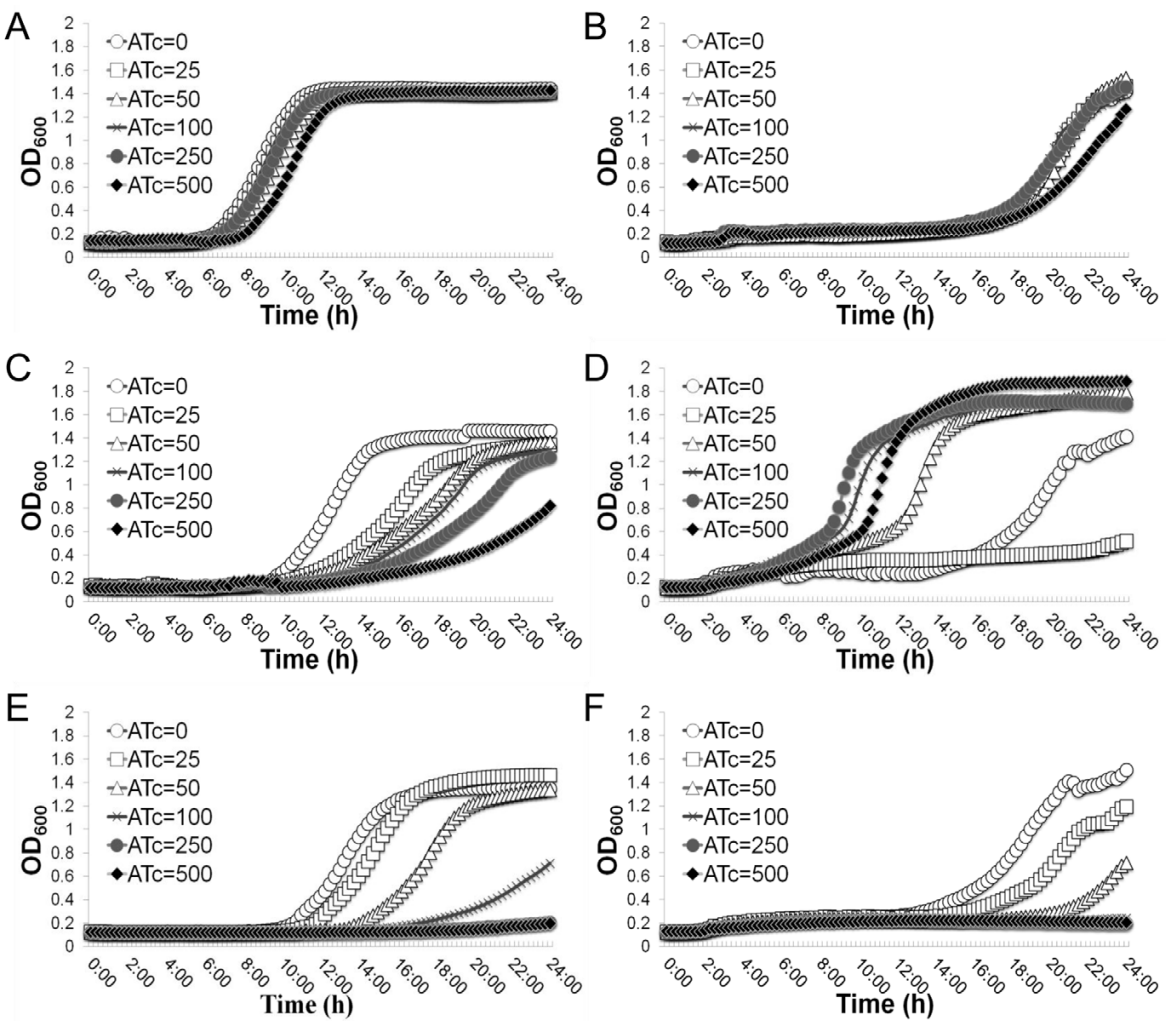
Effect of the depletion of the branched-chain amino acids isoleucine, leucine and valine (ILV) on the growth of the TetR-regulated *gcp* antisense RNA strain. The growth curves of control strain WCUH29 carrying parental plasmid pYH4 in nutrient complete CDM (A) and in ILV dropout CDM (B); the growth curves of the TetR-regulated *gcp* antisense RNA strain (WCUH29/*gcp-as*) in nutrient complete CDM (C) and in ILV dropout CDM (D); and the growth curves of the TetR-regulated *eno* antisense RNA strain (WCUH29/*eno-as* ) in nutrient complete CDM (E) and in ILV dropout CDM (F). The growth curves are monitored by kinetically measuring the optical density at OD600 nm in the corresponding culture medium in the presence of different concentrations of inducer, anhydrotetracyclin (ATc: 0, 25, 50, 100, 250 and 500 ng/ml) every 15 min for 24 hours at 37°C using a BioTek Synergy plate reader. These figures are one representative of three independent experiments.

To eliminate potential effects of isoleucine, leucine and valine amino acids on the regulatory function of the *Pspac*-regulated *gcp* expression system, we confirmed the effect of the depletion of Gcp on growth in ILV dropout CDM using a TetR-regulated *gcp* antisense RNA expression system [4]. The wild-type strain carrying parental plasmid and a TetR-regulated *eno* antisense RNA expression mutant [24] were used as controls. In nutrient complete CDM, the addition of inducer, ATc, had no obvious impact on the growth of the wild-type control strain (Fig. 6A); whereas, the growth of the TetR-regulated *gcp* antisense RNA mutant exhibited an ATc dependent inhibition; and the growth was abolished with a higher dose inducer, 500 µM ATc, indicating the essentiality of Gcp (Fig. 6C). In ILV dropout CDM, the addition of ATc had no obvious influence on growth of the wild-type control (Fig. 6B); whereas remarkably the induced *gcp* antisense RNA enhanced the growth of the TetR-regulated *gcp* antisense RNA strain, when 50 µM or more of inducer ATc was present, indicating the stimulatory effect on growth by the down-regulation of Gcp (Fig. 6D). However, the growth of the *gcp* antisense RNA mutant was dramatically inhibited in the presence of 25 µM ATc compared without inducer, indicating the importance of Gcp for growth (Fig. 5D). In addition, in both nutrient complete CDM and ILV dropout CDM, the growth of the *eno* antisense RNA control strain was remarkably inhibited by the addition of inducer ATc in a dose dependent manner (Fig. 6E, F). These results eliminated the possibility of non-specific effect of ILV dropout on the *Pspac*-regulatory system and the TetR-regulated antisense RNA expression system, and demonstrated that moderate depletion of Gcp has a stimulatory effect on bacterial growth in the absence of BCAAs.

### Modulation of *ilv*-*leu* Operon by Gcp has no Involvement in Gcp’s Essentiality

**Figure 7:**
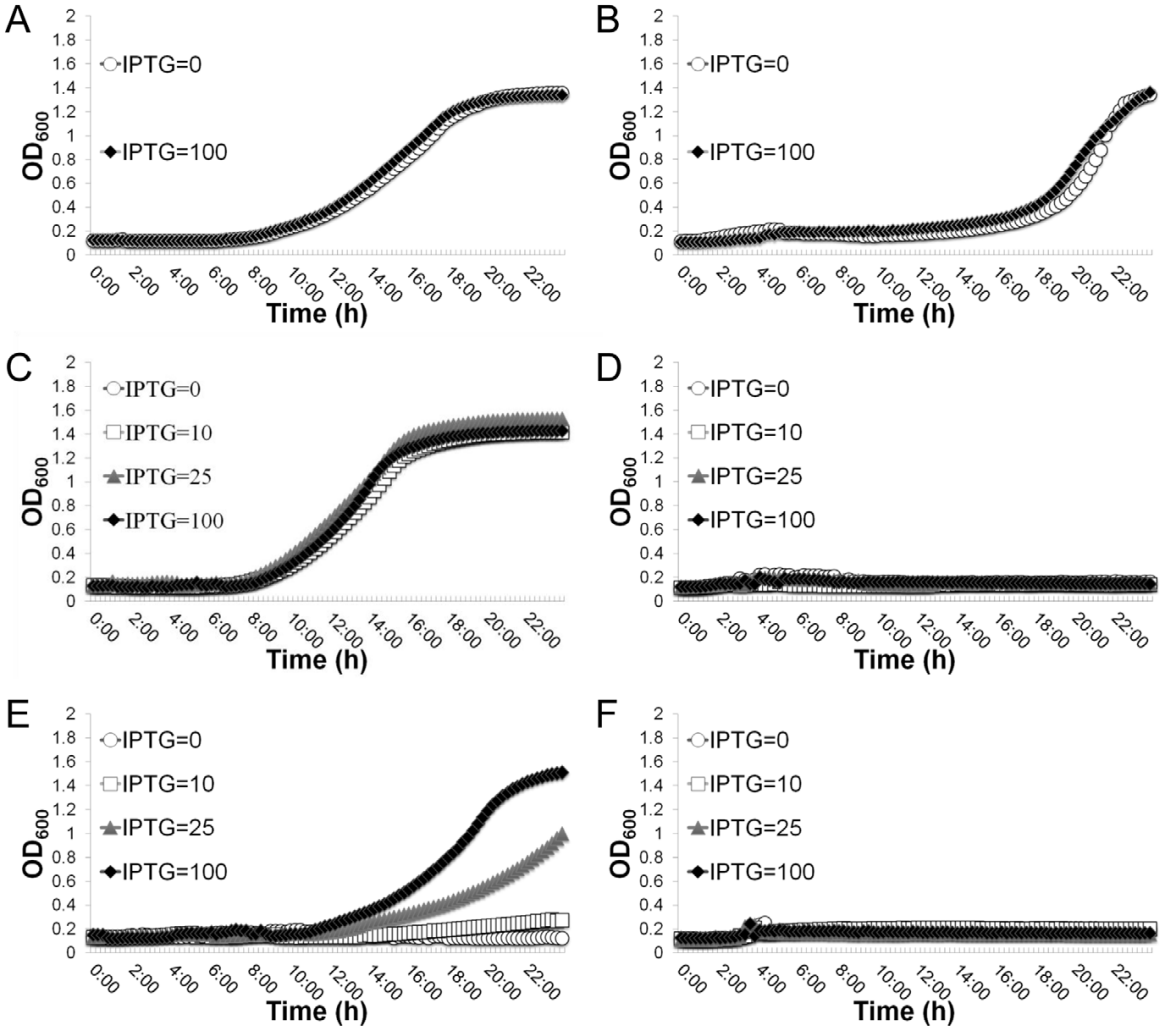
Effect of the deletion of the *ilv*-*leu* operon on the essentiality of Gcp for growth. The growth curves of wild-type control, WCUH29 carrying pYH4-lacI, in nutrient complete CDM (A) and in ILV dropout CDM (B). The growth curves of the *ilv*-*leu* operon deletion mutant (JW290211) in nutrient complete CDM (C) and in ILV dropout CDM (D); the growth curves of the *ilv*-*leu* operon deletion and defined *Pspac*-regulated *gcp* expression strain (JW290311) in nutrient complete CDM (E) and in ILV dropout CDM (F). The growth curves are monitored by kinetically measuring the optical density at OD600 nm in the corresponding culture medium in the presence of different concentrations of inducer, IPTG (0. 10, 25 and 100 µM), in every 15 min at 37°C using a BioTek Synergy plate reader. These figures are one representative of three independent experiments.

The above results suggested that the inhibitory effect on growth by the depletion of Gcp may result from the accumulation of ILV due to the over-expression of the *ilv*-*leu* operon. To test this possibility, we constructed *ilv*-*leu* operon deletion mutants in both the wild-type WCUH29 strain and the *Pspac*-regulated *gcp* expression mutant and examined the effect on the essentiality of Gcp. As expected, in ILV dropout CDM the wild-type control grew slower than that in nutrient complete CDM as expected (Fig. 7A, B). The only deletion of the *ilv*-*leu* operon had no influence on bacterial growth in nutrient complete CDM (Fig. 7C); whereas in ILV dropout CDM the growth of the *ilv*-*leu* null mutants was completely eliminated (Fig. 7D, F). These results further confirmed the null mutation of the *ilv*-*leu* operon by the deletion mutagenesis. Surprisingly, in nutrient complete CDM the deletion of the *ilv*-*leu* operon had no impact on IPTG-dependent growth of the defined *Pspac*-regulated *gcp* expression mutant (Fig. 7E). This result indicates that Gcp’s influence on ILV biosynthesis is not associated with the essential mechanism of Gcp for growth.

## Discussion

The staphylococcal protein Gcp is conserved, belongs to the Kae1/OSGEP/Gcp family [25], and is essential for bacterial growth in a variety species [26]; however, the essential biological function of Gcp remains largely undefined. This study provides direct evidence that the essential protein Gcp is involved in the t6A modification pathway, and negatively regulates the expression of the *ilv*-*leu* operon responsible for the biosynthesis pathway of the branched-chain amino acids (BCAAs): isoleucine, leucine and valine (ILV). In addition, our results showed that the depletion of Gcp dramatically enhanced bacterial growth in chemically defined medium lacking these branched-chain amino acids. Importantly, we demonstrated that the essentiality of Gcp for growth is not attributable to the modulation of the *ilv*-*leu* operon. These findings provide new insights into the biological function of the essential protein Gcp of *S. aureus*.

In this study, we identified significantly altered expression of three key enzymes of the BCAAs biosynthesis pathway, including threonine dehydratase, acetolactate synthase, and dihydroxy-acid dehydratase, during the depletion of Gcp using 2D-DIGE combined with mass spectrometry. These enzymes are encoded by *ilvA*, *ilvB* and *ilvD* gene, respectively, located in an *ilv*-*leu* operon. Although *ilvA*, *ilvB*, and *ilvD* are located in the same *ilv*-*leu* operon, we observed different expression levels of these proteins in 2D-DIGE. A possible explanation for this is that one identified protein spot on 2D-gel may contain several different proteins, although we could not determine the identities of these proteins due to limited peptides coverage and the amount of protein. Another possibility is that the protein levels for each gene are regulated at a post-translational level and could not be determined by our present experiments. Our qPCR and *ilv* promoter-*lux* reporter experiments further confirmed the 2D-DIGE and mass spectrometry results and indicated the transcriptional modulation of *ilv*-*leu* operon by Gcp in *S. aureus*, which is consistent with previous report that the depletion of Gcp homolog, YgjD, in *E. coli* increases the transcription of the *thr* and *ilv* operons [27]. Our qPCR assays revealed that Gcp has a similar influence on the transcriptional level of *ilvD*, *leuA*, and *ilvA*, but has no impact on *ilvE*, which lies outside of the *ilv*-*leu* operon in the *S. aureus* chromosome. These results further indicate the specific effect of Gcp on the *ilv*-*leu* operon that is important for the biosynthesis of BCAAs in *S. aureus*. Although our qPCR and promoter-*lux* reporter results indicated that the transcription of the *ilv*-*leu* operon is highly elevated during the depletion of Gcp, the protein-DNA binding experiment did not reveal any evidence of Gcp binding to the *ilv* promoter region, indicating indirect transcriptional regulation by Gcp. This is consistent with our structural analysis showing that Gcp is unlikely to be a DNA binding protein due to the absence of a DNA binding domain (data not shown). However, this finding is inconsistent with a previous report about a Gcp *Pyrococcus abyssi* homolog, Pa-Kae1, which possesses a novel iron/ATP binding site, as well as DNA binding and apurinic nuclease activity [28]. Taken together, our results indicate an important role of Gcp in the regulation of branched-chain amino acids biosynthesis in *S. aureus*.

Our finding that the staphylococcal Gcp is involved in the t6A modification of tRNA is consistent with recent reports regarding the function of Kae1/Ori7/YgjD in the biosynthesis of N^6^-threonylcarbamoyladenosine (t6A) [29], [30], which is important for the recognition of Ile tRNA synthetases and the formation of charged Ile tRNA [31]. This new finding indicates one conserved function between the staphylococcal Gcp and the *E. coli* homolog YjgD. It is possible that Gcp mediates the transcription of the *ilv*-*leu* operon through the t6A modification pathway, as uncharged tRNA act as a positive regulatory factor to increase gene expression during amino acid limitation [32] and the lack of t6A modification may interfere with T-box dependent regulation of the *ilv* operon [27]. In addition, qPCR revealed that during the depletion of Gcp the increased transcription levels of *ilvD*, *leuA* and *ilvA* genes in one polycistronic operon exhibited significant variations. This suggests that Gcp may affect the transcript processing of the *ilv*-*leu* operon. In addition, we cannot exclude the possibility that in *S. aureus* Gcp may mediates the *ilv-leu* operon at a posttranscriptional level, because in *B. subtilis* it has been revealed that posttranscriptional regulation of the *ilv*-*leu* operon by endoribonuclease and exoribonuclease proteins leads to three different of mRNA transcript lengths of the *ilv*-*leu* operon with varied half life [33].

The mechanism of mediating BCAAs biosynthesis by Gcp is different from other known regulators, including CodY, TnrA, and CcpA, that directly regulate the transcription of *ilv*-*leu* operon by specifically binding to a promoter region [18]. We found that the depletion of Gcp had no influence on *codY* transcription, but increased the *ccpA* transcription by 2-fold, suggesting that Gcp may mediate the expression of *ilv*-*leu* operon, at least in part through the modulation of the positive regulator, CcpA. We are currently working to further determine the impact of CcpA and CodY on Gcp’s modulation of *ilv*-*leu* operon. It is possible that Gcp may control the transcription of *ilv*-*leu* operon through interaction with YeaZ, as the staphylococcal Gcp binds to YeaZ, another essential protein [26]. In *E. coli*, it has been revealed that the Gcp homolog YgjD interacts with YeaZ, which is essential for bacteria growth [34]. Investigation is also underway to determine the impact of YeaZ on Gcp’s involvement in the modulation of BCAAs biosynthesis in *S. aureus*.

Although *S. aureus* maintains a complete set of genes for the biosynthesis of BCAAs the existence of a BCAA auxotrophic phenotype in *S. aureus* is still unclear [20], [35]. In this study, we revealed that moderate depletion of Gcp remarkably enhances bacterial growth in the medium lacking ILV. The ability to stimulate bacterial growth by depleting Gcp in ILV dropout CDM may result from the up-regulation of the *ilv*-*leu* operon and subsequently increase the biosynthesis necessary metabolites and ILV. The essential nature of Gcp requires a basal level expression for proper bacterial cell growth and viability, but at the same time, this expression of Gcp constitutively inhibits ILV biosynthesis. Thus, the BCAA auxotrophic phenotype is likely due to the absence of environmental ILV as well as repression of the intracellular ILV biosynthetic pathway by Gcp, which are important for protein function and bacterial physiology.

The observation that the deletion of the *ilv*-*leu* operon had no impact on the essentiality of Gcp for growth, indicating the repression of ILV biosynthesis pathway is one aspect of the biological function of Gcp, but is not involved in the essential mechanism of Gcp. However, in the absence of exogenous ILV, it seems that Gcp is not required for bacterial growth. The growth of the defined *Pspac*-regulated *gcp* expression mutant and the TetR-regulated *gcp* antisense RNA mutant strains in ILV dropout CDM, compared to wild-type controls, was enhanced when Gcp expression was down-regulated. The growth of these conditional *gcp* mutant strains in ILV dropout CDM depends on the expression of Gcp as well as the biosynthesis of ILV. In our experiments, we observed that the initial inoculum of the defined *Pspac*-regulated *gcp* mutant couldn’t be over-diluted in ILV dropout CDM (we used 1:200 inoculum of overnight culture in CDM) allowing the cells to utilize residual ILV for survival. At a 1:200 dilution, the requirement of Gcp for growth is not titratable due to the carryover of sufficiently expressed Gcp in inoculated bacterial cells compared with those in 1:10^5^–10^6^ diluted cells that are routinely used in down-regulated gene expression studies to determine the essentiality of Gcp. Moreover, our results indicate that the expression levels of Gcp play critical roles in bacterial growth. The lower level of Gcp is important for bacterial growth especially in the medium lacking isoleucine, leucine and valine, because the partial depletion of Gcp can activate the biosynthesis pathway of BCAAs and compensate for the growth defect due to the lack of ILV. In contrast, the high expression levels of Gcp alleviate bacterial growth due to its inhibition of the BCAA biosynthetic pathway, thereafter interfering with bacterial growth in the culture media lacking these essential metabolites.

In conclusion, we have demonstrated that the essential staphylococcal Gcp is involved in the biosynthesis of N^6^-threonylcarbamoyladenosine (t6A), and the repression of the branched-chain amino acids biosynthesis pathway. More importantly, our data indicate that the essentiality of Gcp is independent of its modulation of branched-chain amino acids biosynthesis. These findings provide new insights into the biological function of the staphylococcal Gcp.

## Materials and Methods

### Bacterial Strains, Plasmids, and Growth Conditions

The strains and plasmids used in this study are listed in table 2. *E. coli* strain DH10B was used for plasmid constructions. Luria-Bertani (LB) liquid medium and LB-agar plates were used for the growth and maintenance of *E. coli*. *S. aureus* laboratory strain RN4220 was used as an intermediate host strain prior to introducing plasmids into wild type *S. aureus* strains. *S. aureus* WCUH29 is a clinical isolate that was used for genetic manipulation and growth characterization. Tryptic soy broth (TSB) medium and chemically defined media (CDM) were used for the cultivation of *S. aureus*. Glucose was used as a carbon source at a concentration of 56 mM (1%, w/v). All amino acids included in CDM were L-amino acids. When necessary, isoleucine, leucine and valine were left out of the medium during preparation resulting in ILV dropout CDM.

**Table 2:** Strains and plasmids used in this study.

Strain or plasmid	Description	Reference
Strains		
RN4220	Laboratory strain; *rsbU^-^*	[44]
WCUH29	Clinical human isolate; *rsbU* ^+^	[45]
JRN0105	RN4220::*Pspac*-*gcp*, *spac* promoter regulated *gcp* expression mutant	[3]
JRN0110	JRN0105 with plasmid plux- *P_ilv_*	This study
JW290111	WCUH29 Δ*gcp* attB:: *Pspac*-*gcp* containing plasmid pYH4-lacI	This study
JW290211	WCUH29 Δ*ilv*-*leu* containing plasmid pYH4-lacI	This study
JW290311	WCUH29 Δ*ilv*-*leu* Δ*gcp* attB:: *Pspac*-*gcp* containing plasmid pYH4-lacI	This study
WCUH29/gcp-as	WCUH29 containing TetR regulated *gcp* antisense expression plasmid pYH4/*gcp*-as	[4]
WCUH29/eno-as	WCUH29 containing *TetR* regulated *eno* antisense expression plasmid pYH4/*eno*-as	[24]
Plasmids		
plux-P_ilv_	*ilv* promoter fused with *luxABCDE* on pFF40 [38]	This study
pYH4-lacI	*lacI* gene inserted in the vector pYH4 at *Eco*R I site	This study
pLH1	The integration vector pLL39 [39] carrying *Pspac*-*lacI* segment [46]	This study
pLH1/gcp	pLH1 vector carrying *gcp* gene under regulation of *Pspac* promoter	This study
pYH4/gcp-as	pYH4 vector carrying a *gcp* fragment in an antisense orientation	[3]
pKOR1	*E.coli*/ *S.aureus* shuttle vector, permits lambda recombination and *ccdB* selection;temperature sensitive in *S. aureus*,	[41]
pKOR1/Δgcp	pKOR1 vector carrying *gcp* flanking sequences for *gcp* allelic replacement	This study
pKOR1/Δilv-leu	pKOR1 vector carrying operon *ilv*-*leu* flanking sequences for operon *ilv*-*leu* allelicreplacement	This study

### Two-dimensional Differential in Gel Electrophoresis (DIGE)

Sample preparation was carried out as follows: Briefly, the bacterial cells of mid-log phase culture in TSB in the present or absent IPTG were harvested by centrifugation, placed in lysis buffer (30 mM Tris (pH 8.5), 7 M urea, 2 M thiourea and 4% (w/v) CHAPS), and sonicated on ice. Samples were cleared of insoluble material by centrifugation and quantitated using the Advanced Protein Assay Reagent (Cytoskeleton Denver, CO). Proteins were then subjected to Amersham BioScience CyDye™ minimal labeling per manufacturer’s instructions. Cy2 was designated for labeling a pooled internal standard composed of equal quantities of all samples [36].

2D-DIGE comprised two treatments repeated in triplicate that were co-resolved in three DIGE gels with spots coordinated by the pooled internal standard. Cy2, Cy3 and Cy5 labeled sample was combined for each replicate to 250 µl final volume with immobilized pH gradient (IPG) running buffer (7 M urea, 2 M thiourea, trace bromophenol blue) with DTT and IPG buffer (Amersham Bioscience; pH range same as strip) added to a final concentration of 12 mM and 0.5% v/v, respectively. The samples were allowed to rehydrate into an Immobline DryStrip, pH 4–7, 13 cm strip (GE Healthcare) overnight under low current (30 V for 7 h followed by 60 V for 7 h). After overnight rehydration the samples were resolved with a voltage ramp of 500 V for 0.5 kVh, 1000 V for 0.8 kVh and 8000 V for 17.3 kVh in the Amersham Ettan™ IPGphor ™IEF until a total 19.255 kVh was reached. Strips were equilibrated for 0.5 h in SDS equilibration buffer (50 mM Tris pH 8.8, 6 M urea, 30% glycerol, 4% SDS, 1% DTT) followed by resolution on 8-16% SDS-PAGE BioRad Criterion™ gels. Gels were visualized utilizing the FujiFilm FLA-5000 with the following settings (Cy2: Ex 473 nm, Emission filter 530DF20, Cy3: Excitation 532 nm, Emission filter 570DF20, Cy5: Excitation 635 nm, Emission filter R665). Differential expression between treatments was analyzed using the DeCyder^TM^ version 5.02 (Amersham Bioscience) software package. Spots with an average ratio change of at least 2.5 and a Student’s t-test score of at least 0.05 were deemed of interest.

Protein spots of interest were picked from 2D gels after visualizing with Deep Purple Total Protein Stain (GE Healthcare). Protein spots of interest were robotically excised from the gels using the Genomic Solution™ Investigator ProPic™, and subjected to enzymatic digestion [37] on the Genomic Solution™ ProPrep™, and followed by MALDI mass spectrometry analysis. The identity of identified proteins was revealed using Scaffold and BLAST analysis.

### Construction of a Defined *Pspac*-regulated *gcp* Expression Mutant in *S. aureus*

The construction of a defined *Pspac*-regulated *gcp* expression mutant strain in WCUH29 was performed as described [38]. Briefly, the *gcp* gene was obtained by PCR with primers listed in Table 3, cloned downstream of *Pspac* promoter in the integration vector pLH1 (Table 2) with tetracycline for selection. The recombinant plasmid was electroporated into RN4220 and was integrated into the chromosome at the Phage 11 *attB* site as previously described [39]. The chromosome segment containing the *Pspac*-*gcp* cassette was then transferred from RN4220 to WCUH29 by phage transduction as described using tetracycline selection [40] and confirmed by diagnostic PCR. After the *Pspac*-*gcp* segment was transduced into the WCUH29 chromosome, the endogenous *gcp* gene was then deleted by homogenous recombination using the temperature sensitive plasmid pKOR1 as described [41]. Using the same strategy, we created the *ilv*-*leu* operon deletion mutants in both the defined *Pspac*-regulated *gcp* expression mutant and the parental wild-type WCUH29 strain. The *ilv*-*leu* operon is a 10 kb chromosome segment; to increase the efficiency of allelic replacement events, we deleted the gene cassettes *ilvDBHC* and *leuABCDilvA* sequentially in-frame.

**Table 3:** Primers used in this study.

Name	Sequence
LacIForEcoRI	GCGAATTCTCATCATTTCCTTCCGAAAAAACG
LacIRevEcoRI	GCGAATTCTCACTGCCCGCTTTCCAGTC
PspacLacIForPstI	TTCTGCAGATCTGGTAATGACTCT
PspacLacIRev	TTAGATCTTCACTGCCCGCTTTCCAGTC
GcpLattBFor	BGGGGACAAGTTTGTACAAAAAAGCAGGCTATGGATTGTTATCGCTTAG
GcpLRev	TTCACCCACATAACCATTG
GcpRFor*	AATAAACGTCAGTTATTAACATG
GcpRattBRev	GGGGACAAGTTTGTACAAAAAAGCAGGCTTTAGGAATGTAAAATACGCC
IlvDLattBFor	GGGGACAAGTTTGTACAAAAAAGCAGGCTCGCATCTAATTGCTGTTTAGC
IlvDLRev	AGTAAATTCCCCCGTAAATTTTAATG
IlvCRfor*	GATAGACCTACAATGAGGAGTTG
IlvCRattBRev	GGGGACCACTTTGTACAAGAAAGCTGGGTTTTGTCCGCAATGGTCTTG
LeuALattBFor	GGGGACAAGTTTGTACAAAAAAGCAGGCTCCATAAACCTAAATAACCAATAATTTG
IlvARFor*	CACATAGTAAGAAAAACAGTCATAAATTG
IlvARattBRev	GGGGACCACTTTGTACAAGAAAGCTGGGTCTGTCCTTAGTACGAGAGGACCG
CodYRTFor	GCGCGCGATAAAGCTGCTATTACA
CodYRTRev	ATTAATAGGCCTTCCGTACCGCCA
CcpARTFor	GCCACAGTGTCGCGTGTTGTTAAT
CcpARTRev	ACCTCTAGCAACAGCATTTGGACG
LeuARTFor	CGGCCTTCAAAGTGCTGTTGTTGT
LeuARTRev	ACTTCTGCTTGGGCATCAGTACCT
IlvARTFor	ATTTGTGGACGGTGCATCTGTAGC
IlvARTRev	AAGAGCACTCACACTTAATGCGCC
IlvERTFor	GGCGTTGGTGCATCACATCAGTAT
IlvERTRev	CCACGAACAGCACGCACATATTCA
IlvDRTFor	CACCCGGTATGATTTAGCAG
IlvDRTRev	ACAAGTAGGGCAGGCATTTTG
IlvDproFor	ATCCATTGTTCAATCGTATC
IlvDproRev	GTAAATTTTAATGATTAATCATGTTTTATAG

* :5-terminal end phosphorylated.

### RNA Isolation and Purification

Overnight cultures of *S. aureus* were inoculated at 1% in TSB medium and grown to the mid-exponential (∼4 h) phase of growth. Total RNA was purified from the above cultures, as described (11). Briefly, bacterial cells were harvested by centrifugation at 4,000×g, and the RNA was isolated using the SV total RNA isolation system (Promega). Contaminating DNA was removed with two rounds of DNase treatment (TURBO DNA-*free* kit,Ambion), and the RNA yield was determined spectrophotometrically at 260 nm.

### Semi-quantitative Real-time RT-PCR (qPCR) Analysis

In order to determine the effect of Gcp on the expression of *ilv*-*leu* operon, we employed qPCR to compare the RNA levels as described (11, 16). The first strand cDNA was synthesized using reverse transcriptase SuperScript III and random primers (Invitrogen). For each RNA sample, we performed duplicate reactions of reverse transcription, as well as a control without reverse transcriptase, in order to determine the levels of DNA contamination. PCR reactions were set up in duplicate by using a SYBR Green PCR Master Mix (USB). Real-time sequence-specific detection and relative quantitation were performed with the Stratagene Mx3000P Real Time PCR System. Gene-specific primers were designed to yield 100∼200 bp specific product (Table 3). Relative quantification of the product was calculated using the Comparative C_T_ method, as described for the Stratagene Mx3000P system. The housekeeping gene 16S rRNA was used as an endogenous control (16).

### Construction of *ilvD* Promoter-*lux* Reporter Fusion System

To confirm whether Gcp mediates the transcription of the *ilv*-*leu* operon, we constructed an *ilv* promoter-*lux* reporter fusion system as described [23]. The *ilv* promoter region (25) was amplified by PCR, ligated upstream of promoterless *luxABCDE* in pFF40 [38] resulting in an *ilv*-promoter-*lux* fusion plasmid. The *ilv* promoter-*lux* fusion plasmid was electroporated into the *Pspac-*regulated *gcp* expression strain [3]. Both bioluminescence signals and cell growth were monitored at 37°C by measuring the bioluminescent intensity and optical density at 600 nm with a BioTek Synergy Microplate Reader. To eliminate the effect of bacterial growth, the relative light units (RLU) were calculated (light intensity/OD_600_) from triplicate readings at different times during growth.

### Gel Mobility Shift DNA Binding Assay

To determine whether Gcp directly regulates the transcription of the *ilv*-*leu* operon, we performed gel-shift assays. The DNA fragment of the upstream region of *ilv* was obtained by PCR using the primers listed in Table 3. The amplified DNA fragment was purified and labeled with Digoxigenin using a DIG Gel Shift kit (Roche) according to the manufacturer’s protocol. The DNA-binding and electrophoresis were performed, as described (17, 20). Briefly, the purified PCR products were labeled with Digoxigenin using terminal transferase (Roche). The labeled DNA fragments were further purified to remove the redundant DIG-ddUTP and salts. The interaction of Gcp with DNA was conducted in a 20 μl reaction mixture containing 0.2 pmol DIG-labeled DNA, 1 μg of poly-[d(I–C)], 25 mM NaH_2_PO_4_ (pH 8.0), 50 mM NaCl, 2 mM MgCl_2,_ 1 mM DTT, 10% glycerol, 0.1 mM EDTA, and different concentrations of Gcp protein. Unlabeled DNA fragments of the promoter region as a specific competitor were added into the reaction with 100-fold excess to labeled probe. Internal gene fragments were obtained by PCR, purified, and labeled as nonspecific controls. BSA was used as a nonspecific protein binding control. The DNA binding reaction was initiated by the addition of Gcp and incubated at room temperature for 25 min. Samples were then loaded directly onto a 5% native polyacrylamide gel [acrylamide:bisacrylamide (29:1) in 0.5×TBE buffer]. Electrophoresis was run for 2 h at 4°C with 7 V/cm, and the gels were transferred to Nylon membrane via electro-blotting in 0.5×TBE at 300 mA for 90 min. at 4°C. After cross-linking of DNA fragments using UV, the membranes were hybridized with anti-digoxigenin-AP antibody and exposed to X-ray film for 4 h to achieve the desired signal.

### Purification of Bulk tRNA

The conditional *S. aureus* cells were grown in 100 ml TSB media at 37°C supplemented with or without 200 µM IPTG to OD_600_ = 0.4, harvested, and washed in cold water. The bacterial cells were broken using a Bead Beater as described [42]. Briefly, the bacterial cells were resuspended in 1.5 ml DEPC-treated water and were then transferred into two 2 ml Eppendorf tubes containing equal volume (750 µl) of 0.1 mm glass beads. The bacterial cells were broken using a Mini-Bead Beater-8 at maximum speed for 4 min at 4°C. The supernatant was transferred into a new 1.5 ml Eppendorf tube; and bulk tRNA was isolated using the mirVana^TM^ miRNA isolation kit (Ambion) according to the manufacturer’s instruction. The tRNA was eluted in water and stored at −80°C. The concentration of tRNA was measured by using a Nano Drop (Thermo Scientific).

### LC-MS/MS Analysis of Digested tRNA

To quantify the t6A content of the tRNA, samples of each purified bulk tRNA were enzymatically hydrolyzed to nucleotides as described [43]. Briefly, 15 µg of bulk tRNA was sequentially digested with 5 units of nuclease P1 (Sigma) at 45°C for 16 h, 0.01 units of Phosphodiesterase I (Sigma) at 37°C for 2 h, and then 10 units of calf intestine alkaline phosphatase (Promega) at 37°C for 1 h in a total volume of 70 µl. The resulting nucleotides were acidified by the addition of 1–5 µl 10% formic acid and monitored using pH indicator paper, clarified by centrifugation, and then purified by passing through a C18 zip tip column (Millipore). The purified samples were dehydrated, suspended in 50 µl of 5 mM ammonium acetate, pH 6.0, and diluted (1 µl to 20 µl). The 20 µl diluted samples were then subjected to injection using an Agilent autosampler LCMSMS with an analytical Waters Symmetry C18, 3.5 µm column connected to the Applied Biosystem 4000 iontrap fitted with a turbo V electrospray source. The samples were subjected to a linear gradient of 5 mM ammonium acetate (pH 6.0) to 100 percent acetonitrile for 10 minutes at a column flow rate of 200 µl /min. Transitions monitored were the m/z 413 > m/z 281 and the m/z 413> m/z 136 for the t6A and m/z 268> m/z 136 for the adenosine. The data was analyzed using MultiQuant (ABI) providing the peak area for the transitions. A standard curve was constructed using concentrations of t6A from fentomole to nanomole in 20 µl.

### Characterization of Bacteria Growth

The *S. aureus* strains were inoculated in TSB, CDM or ILV dropout CDM with appropriate antibiotics and different concentrations of IPTG (0, 10, 25, and 100 μM) or antisense RNA inducer, anhydrotetrocycline (ATc), at 37°C. The bacterial growth was monitored by kinetically measuring optical density at 600 nm with a BioTek Synergy Microplate reader.
